# Monodehydroascorbate reductases in plants: integrating ascorbate recycling, redox signaling, and stress adaptation

**DOI:** 10.1016/j.redox.2026.104382

**Published:** 2026-09-02

**Authors:** Yuxin Xiao, Jiatong Zhou, Shengchun Li

**Affiliations:** School of Life Sciences, Hubei University, Wuhan, 430062, China

**Keywords:** MDHAR, ROS, Ascorbate-glutathione cycle, Redox signaling, Stress adaptation

## Abstract

Reactive oxygen species (ROS) are central regulators of plant growth, development, and environmental adaptation, with hydrogen peroxide (H_2_O_2_) acting as a key signaling molecule. The ascorbate-glutathione cycle is the major antioxidant pathway that maintains H_2_O_2_ homeostasis, and monodehydroascorbate reductase (MDHAR) plays a critical role by regenerating reduced ascorbate (ASC) from monodehydroascorbate (MDHA). Although traditionally viewed as an ASC-recycling enzyme, increasing evidence indicates that MDHAR has broader functions in redox regulation. Recent studies have revealed that MDHAR contributes to stress responses, developmental regulation, and ROS signaling through mechanisms that cannot be fully explained by ASC recycling alone. In this review, we summarize recent advances in the structural characteristics, regulation, subcellular specialization, and evolution of plant MDHARs. We also discuss emerging non-canonical functions of MDHAR, alternative pathways for MDHA reduction, and the mechanistic basis for the contrasting effects of MDHAR manipulation on ASC accumulation. Finally, we propose that MDHAR functions as an integrative hub linking ascorbate metabolism with cellular redox networks and highlight key challenges and future opportunities for exploiting MDHAR to improve crop stress tolerance, productivity, and nutritional quality.

## 1 Introduction

**Fig. 1:**
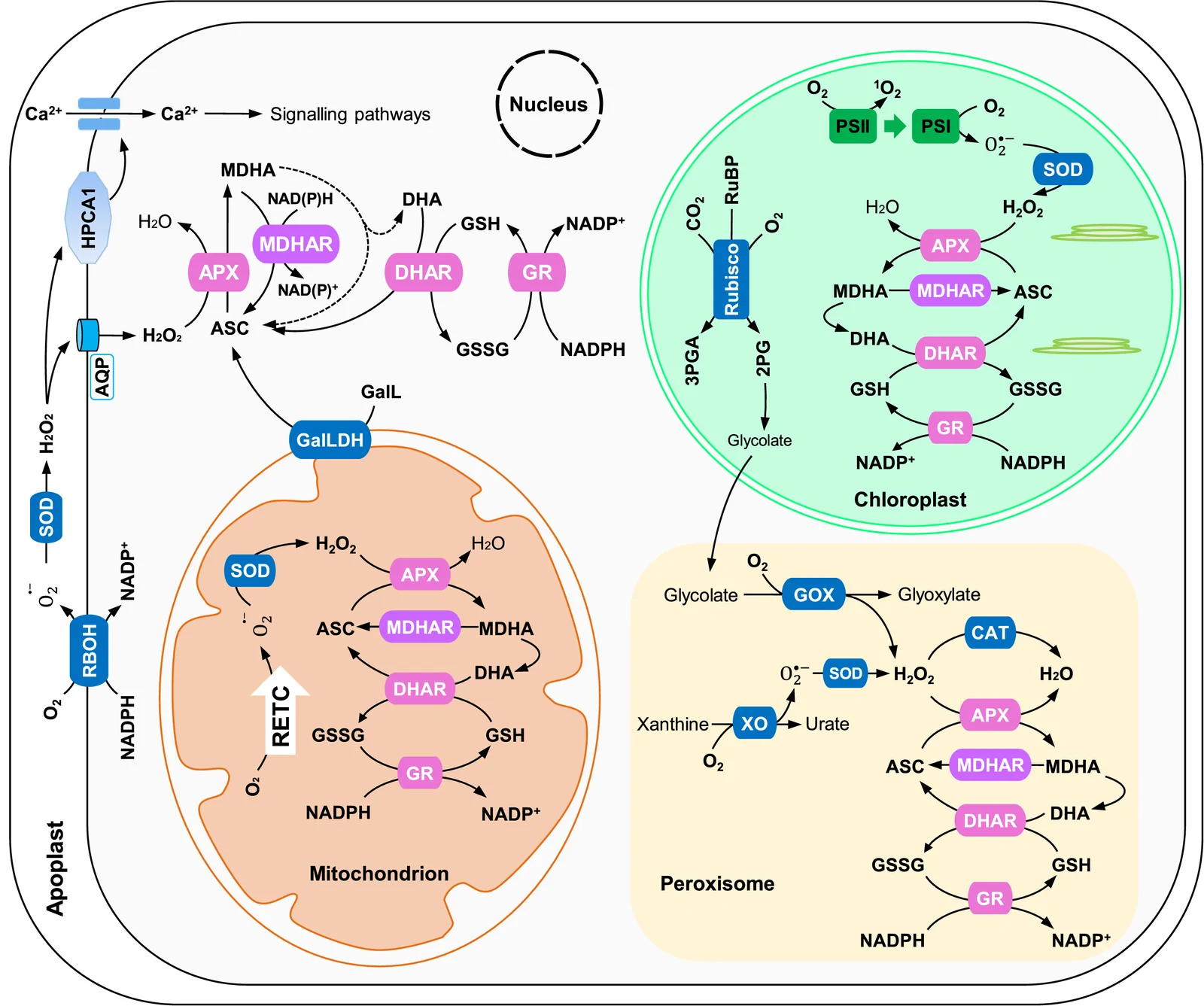
**Basics of ROS formation in plant cells and the critical role of MDHAR in the ascorbate pool.** ROS are continuously generated in multiple subcellular compartments. During photosynthesis, ^1^O_2_ is produced primarily by PSII, whereas $O_{2}^{\cdot -}$ is generated by photosynthetic and mitochondrial electron transport chains under conditions of excess electron flux. H_2_O_2_ is formed through enzymatic or spontaneous dismutation of $O_{2}^{\cdot -}$ and during photorespiration via GOX. H_2_O_2_ can diffuse across biological membranes through aquaporins and functions as an important intracellular signaling molecule. Cellular H_2_O_2_ homeostasis is maintained by the ascorbate–glutathione cycle operating in the cytosol, chloroplasts, mitochondria, and peroxisomes. ASC is synthesized in mitochondria and transported to other cellular compartments. During H_2_O_2_ detoxification, ASC is oxidized by APX to MDHA, which is either reduced back to ASC by MDHAR or spontaneously disproportionates into ASC and DHA. DHA is subsequently regenerated to ASC by DHAR using GSH, whereas GR restores GSH from GSSG. Abbreviations: APX, ascorbate peroxidase; AQP, aquaporin; ASC, reduced ascorbate; DHA(R), dehydroascorbate (reductase); Fd, ferredoxin; GalLDH, l-galactono-1,4-lactone dehydrogenase; GOX, glycolate oxidase; GR, glutathione reductase; GSH, reduced glutathione; GSSG, oxidized glutathione; HPCA1, hydrogen peroxide-induced Ca^2+^ increases 1; MDHA(R), monodehydroascorbate (reductase); PSI/II, photosystem I/II; RBOH, respiratory burst oxidase homolog; RETC, respiratory electron transport chain; Rubisco, ribulose-1,5-bisphosphate carboxylase/oxygenase; RuBP, ribulose-1,5-bisphosphate; SOD, superoxide dismutase; XO, xanthine oxidase; 2 PG, 2-phosphoglycolate; 3PGA, 3-phosphoglycerate.

Reactive oxygen species (ROS) are now recognized as fundamental regulators of plant physiology, integrating environmental signals with growth, development, and stress acclimation [[1], [2], [3], [4]]. Rather than representing merely harmful by-products of aerobic metabolism, ROS function as highly dynamic signaling molecules that coordinate cellular responses to continuously changing environmental conditions. Among the different ROS, hydrogen peroxide (H_2_O_2_) occupies a particularly prominent position because of its relatively long lifetime, ability to diffuse across biological membranes via aquaporins, and perception by dedicated receptor proteins, enabling both local and long-distance signaling [5,6] (Fig. 1). A notable example is the plasma membrane receptor hydrogen-peroxide-induced Ca^2+^ increases 1 (HPCA1), which senses extracellular H_2_O_2_ through redox-sensitive cysteine residues, triggering Ca^2+^ influx and initiating signaling cascades that coordinate plant responses to diverse abiotic and biotic stresses [7,8]. Consequently, maintaining H_2_O_2_ homeostasis is essential not only for preventing oxidative damage but also for preserving the signaling functions of ROS.

Cellular ROS homeostasis is maintained through a dynamic balance between ROS production and antioxidant scavenging. Central to this regulatory network is the ascorbate-glutathione cycle, also known as the Foyer-Halliwell-Asada pathway, which constitutes the major enzymatic system responsible for H_2_O_2_ detoxification in plants [9,10]. During this process, ascorbate peroxidase (APX) reduces H_2_O_2_ to water using reduced ascorbate (ASC), generating the unstable intermediate monodehydroascorbate (MDHA). MDHA is either rapidly recycled to ASC by monodehydroascorbate reductase (MDHAR) using NAD(P)H or spontaneously disproportionates to ASC and dehydroascorbate (DHA). DHA is subsequently reduced to ASC by dehydroascorbate reductase (DHAR) using reduced glutathione (GSH), while glutathione reductase (GR) regenerates GSH from oxidized glutathione (GSSG) at the expense of NADPH (Fig. 1). Through the coordinated activities of these enzymes, the ASC pool is continuously regenerated, thereby sustaining antioxidant capacity while maintaining cellular redox homeostasis.

Among the enzymes of the ascorbate-glutathione cycle, MDHAR has traditionally been regarded as a dedicated ASC-recycling enzyme. However, this view has become increasingly difficult to reconcile with recent experimental evidence. Genetic, biochemical, and physiological studies demonstrate that MDHAR contributes to diverse biological processes, including ROS signaling, stress adaptation, source-sink regulation, and plant development, through mechanisms that extend beyond its canonical role in ASC regeneration. Moreover, manipulation of MDHAR frequently produces contrasting effects on ASC accumulation and stress tolerance across species, tissues, developmental stages, and environmental conditions. These observations challenge the long-standing assumption that MDHAR functions primarily as a determinant of cellular ASC content and instead suggest that it operates within an integrated redox network in which antioxidant metabolism, ROS signaling, and developmental programs are tightly interconnected.

Despite substantial advances in our understanding of the ascorbate-glutathione cycle, comprehensive reviews have focused primarily on other pathway components, including APX [11,12], DHAR [13], GR [14], ascorbate [15,16], and glutathione [17,18]. By contrast, a modern synthesis of MDHAR biology that integrates recent discoveries across molecular, physiological, and evolutionary levels remains lacking. Here, we provide a comprehensive overview of current advances in plant MDHAR research. We examine the structural and biochemical properties, regulatory mechanisms, evolutionary diversification, and subcellular specialization of MDHAR isoforms, and discuss emerging evidence for non-canonical catalytic activities and alternative pathways for MDHA reduction. We further evaluate the mechanistic basis underlying the inconsistent effects of MDHAR manipulation on ASC homeostasis and highlight recent discoveries linking MDHAR to redox signaling, stress adaptation, and developmental regulation. Finally, we propose a conceptual framework in which MDHAR functions as an integrative hub connecting ascorbate metabolism with broader cellular redox networks, and we discuss key knowledge gaps and future directions for exploiting MDHAR in crop improvement.

## 2 Molecular characteristics, regulatory mechanisms, and subcellular compartmentalization of plant MDHAR isoforms

### 2.1 Gene family expansion and subcellular specialization

The *MDHAR* gene family has expanded substantially during plant evolution, reflecting increasing functional complexity of cellular redox regulation. Whereas unicellular green algae typically possess a single *MDHAR* gene, most land plants contain multiple paralogs generated through gene duplication and whole-genome duplication events [19]. Polyploid species exhibit particularly extensive expansion, with 12 *MDHAR* genes identified in allotetraploid cotton (*Gossypium barbadense* and *G. hirsutum*), 15 in allohexaploid wheat (*Triticum aestivum*), and as many as 168 homologs in cultivated octoploid strawberry (*Fragaria × ananassa*) [[20], [21], [22]]. Such dramatic expansion is unlikely to represent simple genetic redundancy and instead suggests progressive functional diversification following gene duplication.

Consistent with this view, MDHAR isoforms are distributed among multiple subcellular compartments, including the cytosol, chloroplasts, mitochondria, and peroxisomes, where they maintain compartment-specific redox homeostasis. In Arabidopsis, five *MDHAR* genes generate isoforms with distinct intracellular localizations. AtMDHAR1 is dual-targeted to the cytosol and peroxisomal matrix through a weak peroxisomal targeting signal [23,24]. AtMDHAR2 and AtMDHAR3 are exclusively cytosolic, whereas AtMDHAR4 is anchored to the peroxisomal membrane through a membrane-specific targeting sequence [23]. A fifth gene produces both chloroplast- and mitochondrion-targeted proteins via alternative transcription initiation; these proteins were originally designated AtMDHAR5 and AtMDHAR6 but are now recognized as products of a single locus [19]. Similar compartmentalized isoform families have been identified in rice (*Oryza sativa*) and maize (*Zea mays*) [25,26], whereas tomato possesses three principal isoforms localized to peroxisomes, chloroplasts, and the cytosol/peroxisomes, respectively [19]. The conserved partitioning of MDHAR among distinct organelles suggests that individual isoforms are adapted to the specific redox environments and metabolic demands of each compartment. Understanding how these spatially separated enzymes cooperate to coordinate whole-cell ASC metabolism and ROS signaling remains a major challenge for future research.

### 2.2 Structural features and cofactor preference

MDHAR is a highly conserved flavin adenine dinucleotide (FAD)-dependent oxidoreductase that is ubiquitously distributed throughout the plant kingdom. The enzyme functions as a monomer and contains catalytically essential thiol residues required for MDHA reduction [20]. Although MDHAR is capable of using either NADH or NADPH as an electron donor, biochemical analyses consistently show that most plant isoforms preferentially utilize NADH [19,[27], [28], [29], [30], [31], [32]]. This preference is maintained even in chloroplast-localized isoforms, where NADPH is considerably more abundant than NADH [33] (Table 1), suggesting that cofactor selectivity reflects an evolutionarily conserved property of the enzyme rather than adaptation to organelle-specific metabolite availability.

**Table 1:** *K*_m_ value for NADH and NADPH of MDHAR from various plant species.

Species	MDHAR	Subcellular localization	*K*_m_ (μm)		Ref
			NADH	NADPH	
Arabidopsis	^R^MDHAR1	Cyt/Per	8.3	217	[30]
	^R^MDHAR2	Cyt	3.5	9.5	
Cucumber	Fruit MDHAR	Unknown	4.6	23	[28]
	^R^MDHAR	Cyt	4.4	210	[29]
Pea	^R^MDHAR	Cyt/Per	5.3	21.5	[31]
Potato	Tuber MDHAR	Cyt	7.7	30	[34]
	Tuber MDHAR	Mit	12.3	57.3	[35]
Soybean	Root nodule MDHAR	Unknown	5.6	150	[36]
Spinach	Leaf MDHAR	Chl	7	22	[27]
	^R^MDHAR	Chl	6.3	430	[33]
*Physcomitrella patens*	^R^MDHAR1	Cyt	7.8	88	[37]
	^R^MDHAR2	Cyt	9.6	223	
	^R^MDHAR3	Cyt	17.8	990	

^R^, recombinant protein expressed in *Escherichia coli*.

Chl, chloroplast; Cyt, cytosol; Mit, mitochondrion; Per, peroxisome.

The crystal structure of rice OsMDHAR has provided important mechanistic insights into substrate recognition and electron transfer [32]. OsMDHAR adopts a structural fold characteristic of iron-sulfur protein reductases and contains an extended loop (residues 63–80) adjacent to the catalytic pocket that is absent from related flavoproteins. Mutational analyses identified Arg320 as a key determinant of MDHA binding, whereas Tyr349 mediates electron transfer from NAD(P)H to MDHA through the FAD cofactor. In addition, a hydrogen bond formed between Glu196 and the adenosine moiety of NADH largely accounts for the enzyme's marked preference for NADH over NADPH [32]. Functional diversification is also evident among individual MDHAR isoforms. In *Arabidopsis thaliana*, the peroxisomal isoform AtMDHAR1 provides most NADH-dependent MDHA reductase activity in leaves, whereas the cytosolic isoform AtMDHAR2 contributes predominantly to NADPH-dependent [30]. These findings indicate that cofactor preference and subcellular localization have co-evolved to generate isoforms with distinct biochemical properties, enabling MDHAR to support redox homeostasis in different cellular compartments.

### 2.3 Multilayered regulation of MDHAR activity

MDHAR activity is regulated at multiple levels, including transcriptional, post-transcriptional, and post-translational mechanisms, allowing ASC recycling to respond rapidly to developmental programs and environmental fluctuations. Promoter analyses have identified numerous cis-regulatory elements associated with light signaling, phytohormones, and abiotic stress responses, indicating that MDHAR expression is integrated into diverse signaling pathways [[20], [21], [22]]. However, relatively few upstream regulators have been experimentally characterized. One validated regulatory module has been described in apple (*Malus domestica*), where the GRAS transcription factor MsSCL26.1 directly activates MDHAR expression to maintain ASC homeostasis, while miR171i represses MsSCL26.1, thereby establishing a transcriptional circuit that fine-tunes MDHAR abundance [38]. Similarly, several microRNAs are predicted to target individual *TaMDHAR* genes in bread wheat, suggesting that post-transcriptional regulation contributes to developmental plasticity and stress adaptation [21,39]. Although these observations support multilayered regulation of *MDHAR* expression, the upstream signaling pathways that coordinate these regulatory networks remain largely unknown.

**Fig. 2:**
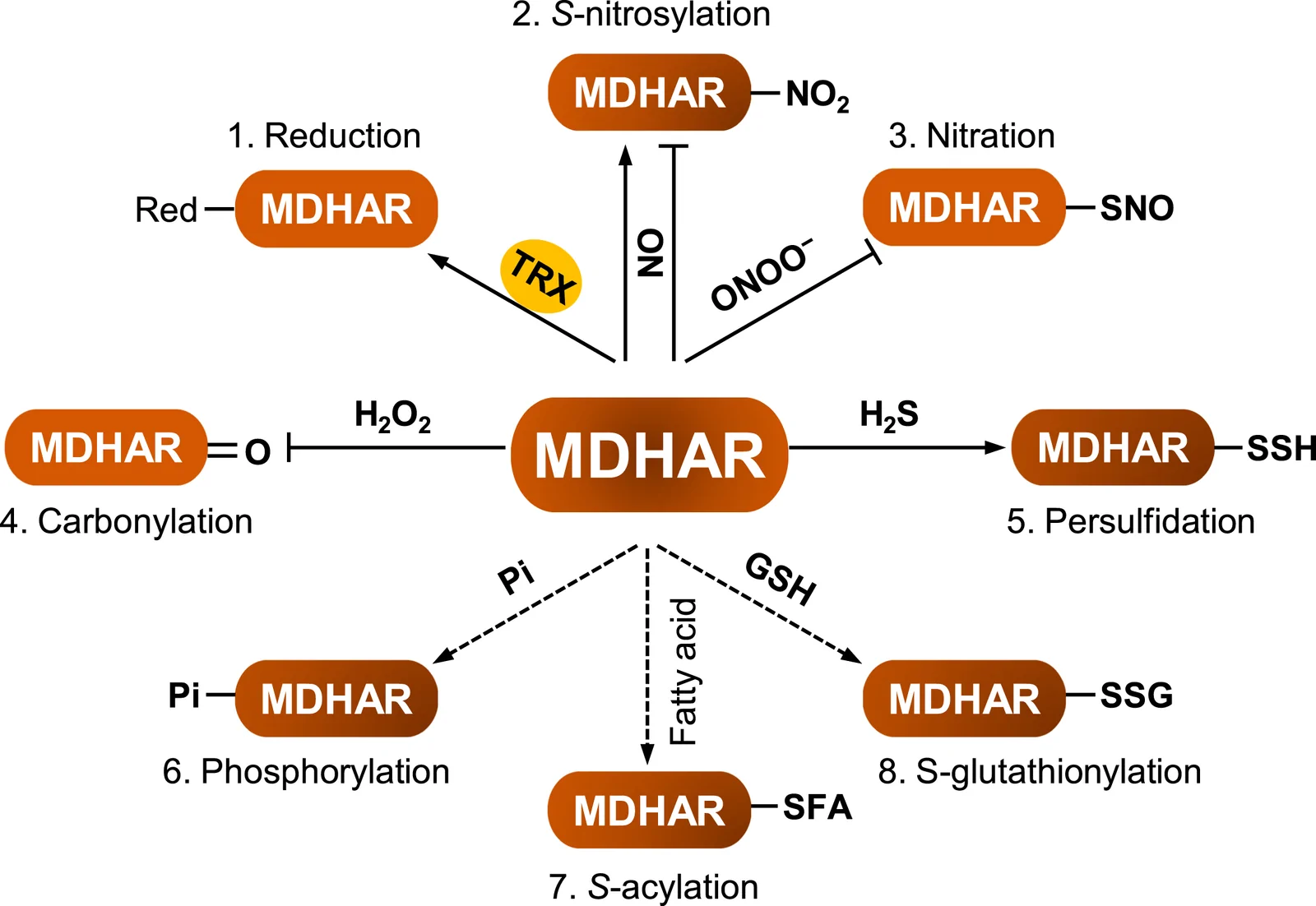
**Post-translational modifications (PTMs) regulating plant MDHAR.** Experimentally reported PTMs of plant MDHAR, including *S*-nitrosylation, tyrosine nitration, carbonylation, phosphorylation, glutathionylation, persulfidation, and *S*-acylation. Solid arrows indicate activation of MDHAR activity, T-shaped lines indicate inhibition, and dashed arrows denote regulatory mechanisms that remain to be experimentally established.

Post-translational modifications (PTMs) provide an additional mechanism for rapidly modulating MDHAR activity in response to changes in cellular redox status (Fig. 2). Among these, thiol-based redox modifications have received the greatest attention. During drought stress, chloroplast-localized AtMDHAR6 is activated by plastidial y-type thioredoxins (TRXs), thereby enhancing NADPH-dependent MDHA reduction and supporting chloroplast redox homeostasis [40]. Nitric oxide (NO)-dependent modifications also exert profound but contrasting effects on MDHAR activity. *S*-nitrosylation has been detected in MDHAR proteins from several plant species, including Arabidopsis, citrus (*Citrus aurantium*), and rice [[41], [42], [43]]. In transgenic tobacco (*Nicotiana tabacum*) expressing tomato (*Solanum lycopersicum*) *SlMDHAR3*, *S*-nitrosylation enhances MDHAR activity and contributes to improved salt tolerance [44]. Likewise, low-temperature stress promotes brassinosteroid-dependent NO production in transgenic Arabidopsis expressing MDHAR from mini Chinese cabbage (*Brassica pekinensis*), low-temperature stress induces brassinosteroid-dependent NO production, leading to *S*-nitrosylation-mediated enzyme activation and enhanced cold tolerance [45]. In contrast, NO-dependent modifications can also suppress MDHAR activity. In pea (*Pisum sativum*), both *S*-nitrosylation and tyrosine nitration inhibit peroxisomal MDHAR, with Tyr345 identified as the major nitration site responsible for enzyme inactivation [46]. These apparently opposing effects indicate that the physiological consequences of NO signaling depend on species, isoform identity, and cellular context rather than representing a universal mode of regulation.

Additional PTMs further expand the regulatory landscape of MDHAR. Oxidative carbonylation completely abolishes the activity of Arabidopsis AtMDHAR4, and its accumulation increases markedly under oxidative stress [47]. Hydrogen sulfide (H_2_S) -dependent persulfidation activates AtMDHAR6, and this modification can be reversed by mitochondrial TRXo1 [48]. Predicted phosphorylation sites are abundant in wheat MDHAR proteins, particularly TaMDHAR2-B, although their biological functions remain unknown [21]. Proteomic studies have also identified *S*-acylation and glutathionylation of Arabidopsis MDHAR proteins [49,50]. Whether these modifications regulate catalytic activity, protein stability, subcellular trafficking, or protein-protein interactions have yet to be determined. Together, current evidence suggests that PTMs provide a dynamic interface through which developmental and environmental signals modulate MDHAR function, but the underlying molecular mechanisms remain largely unresolved.

## 3 Canonical functions of MDHAR and alternative enzymatic pathways mediating MDHA reduction

### 3.1 Broad substrate specificity underpins non-canonical functions of MDHAR

**Fig. 3:**
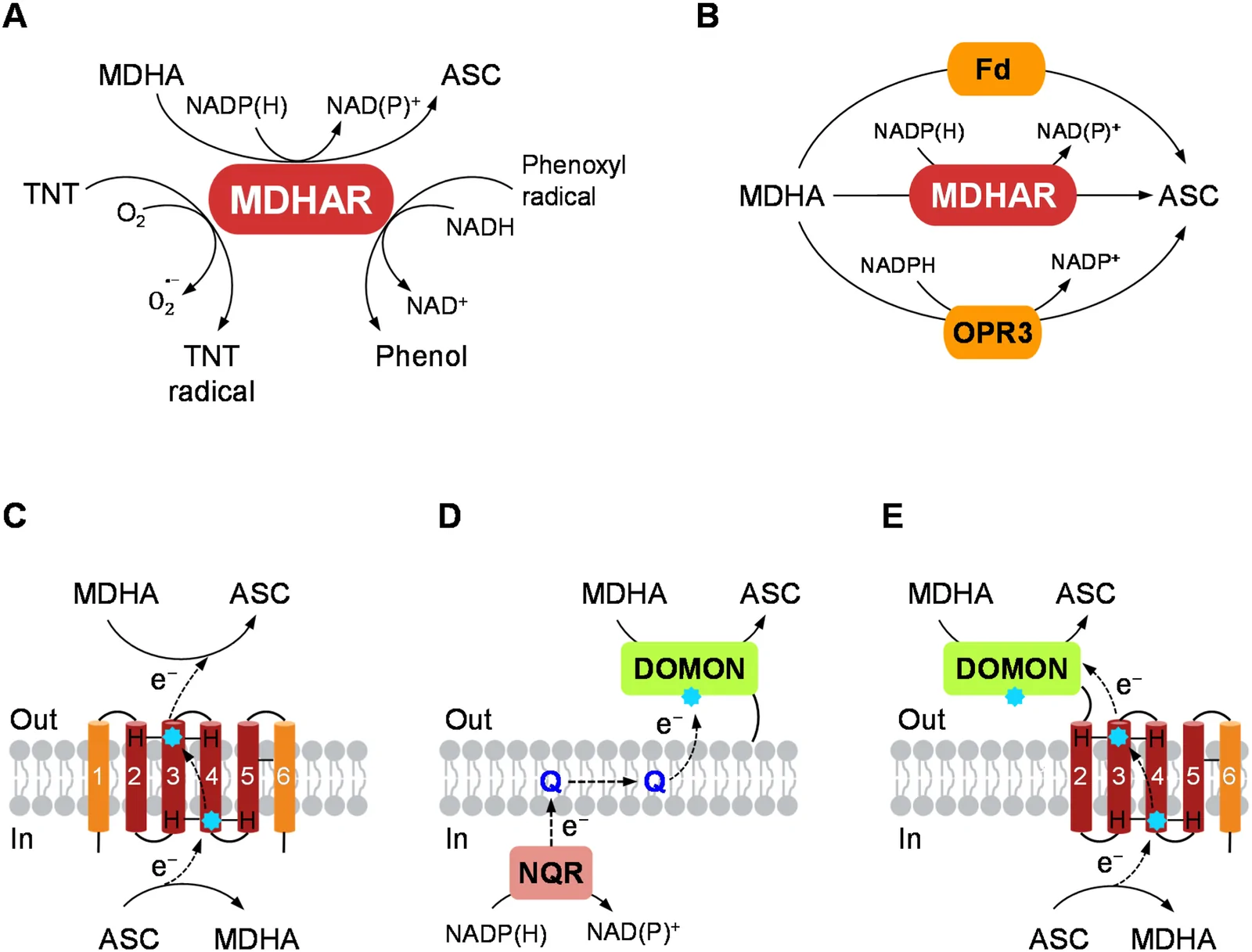
**Non-canonical functions of MDHAR and alternative pathways for MDHA reduction in plants**. **A**. In addition to its canonical substrate MDHA, MDHAR catalyzes the reduction of several non-canonical substrates, including phenoxyl radicals and TNT, indicating broader physiological functions beyond ASC recycling. **B**. Alternative enzymatic pathways for MDHA reduction mediated by ferredoxin (Fd) and 12-oxophytodienoate reductase 3 (OPR3). **C.** Proposed mechanism of MDHA reduction mediated by cytochrome *b*561 (CYB561). CYB561 is an integral membrane protein containing six transmembrane helices and two heme cofactors coordinated by four conserved histidine residues. Electrons derived from cytosolic ASC are transferred across the membrane through the heme groups (shown in cyan) and to reduce extracellular MDHA, thereby regenerating ASC. **D**. Proposed MDHA reduction catalyzed by dopamine β-monooxygenase N-terminal (DOMON)-domain proteins. The Arabidopsis protein AIR12 contains a heme-binding DOMON domain that catalyzes MDHA reduction. A hypothetical model proposes that electrons are transferred from cytosolic NAD(P)H through NAD(P)H quinone oxidoreductase (NQR) and quinone intermediates to support extracellular ASC regeneration. **E**. Proposed MDHA reduction mediated by CYBDOM proteins. CYBDOM proteins combine a CYB561 transmembrane electron-transfer module with one or more extracellular DOMON domains, enabling coordinated transmembrane electron transport for reduction of extracellular MDHA to ASC.

Although MDHAR is best known for catalyzing the reduction of MDHA during ASC recycling, accumulating evidence indicates that it functions as a versatile oxidoreductase with substrate specificities extending well beyond MDHA (Fig. 3A). This catalytic flexibility broadens the physiological roles of MDHAR and suggests that many phenotypes associated with altered MDHAR activity cannot be attributed solely to changes in ASC metabolism. One prominent example is the reduction of phenoxyl radicals derived from diverse phenolic compounds, including quercetin, ferulic acid, coniferyl alcohol, and chlorogenic acid [51]. Through these reactions, MDHAR contributes to the maintenance of phenolic antioxidants and may influence lignification, secondary metabolism, and protection against oxidative stress. More recently, MDHAR has also been implicated in xenobiotic metabolism. In *Arabidopsis*, the mitochondrial isoform AtMDHAR5 interacts with the environmental contaminant 2,4,6-trinitrotoluene (TNT), promoting superoxide ($O_{2}^{\cdot -}$) production and mediating TNT-induced phytotoxicity [52]. These findings demonstrate that MDHAR participates in metabolic processes extending beyond antioxidant recycling and highlight its broader roles in environmental adaptation.

The expanding repertoire of MDHAR substrates has important implications for interpreting genetic studies. Physiological changes observed in MDHAR overexpression or loss-of-function lines may result not only from altered ASC recycling but also from perturbations in phenolic metabolism, xenobiotic detoxification, or other redox-dependent pathways. Moreover, substrate specificity appears to vary among subcellular isoforms, suggesting that functional diversification has accompanied compartmental specialization during evolution. Defining the substrate spectrum of individual MDHAR isoforms therefore represents an important step toward understanding their physiological functions.

### 3.2 Alternative pathways for MDHA reduction

Although MDHAR provides the principal enzymatic route for MDHA reduction, it is not the only mechanism by which ASC is regenerated. Plants possess multiple complementary enzymatic and non-enzymatic pathways that reduce MDHA, collectively forming a robust and highly buffered redox network that maintains ASC homeostasis under diverse physiological conditions.

Within chloroplasts, photoreduced ferredoxin (Fd) generated by photosystem I can reduce MDHA with considerably higher efficiency than NADPH-dependent MDHAR [53] (Fig. 3B). Consequently, Fd-mediated reduction is thought to represent a major pathway for MDHA recycling during photosynthesis, whereas MDHAR plays a more supportive role within the chloroplastic ascorbate-glutathione cycle. In peroxisomes, 12-oxophytodienoic acid reductase 3 (OPR3), an enzyme primarily associated with jasmonic acid biosynthesis, also exhibits NADPH-dependent MDHA reductase activity, thereby linking ASC regeneration with oxylipin metabolism and providing an additional route for maintaining peroxisomal redox balance [54] (Fig. 3B).

Additional MDHA-reducing systems operate at cellular membranes. Cytochrome *b*561 (CYB561) proteins are ASC-dependent transmembrane oxidoreductases localized to the apoplast, tonoplast, and Golgi membranes, where they mediate transmembrane electron transport to regenerate ASC across membrane barriers [[55], [56], [57], [58]] (Fig. 3C). At the plasma membrane, dopamine β-monooxygenase N-terminal (DOMON)-domain proteins, exemplified by AIR12, receive electrons from ASC or $O_{2}^{\cdot -}$ and subsequently transfer them to MDHA or molecular oxygen, thereby contributing to extracellular redox regulation [59,60] (Fig. 3D). More recently, CYBDOM proteins, which combine CYB561 and DOMON domains within a single polypeptide, have been identified as efficient transmembrane electron carriers that couple cytosolic ASC oxidation to apoplastic MDHA reduction [61] (Fig. 3E). These membrane-associated systems extend ASC recycling beyond intracellular compartments and provide an effective mechanism for maintaining extracellular redox homeostasis.

Collectively, these complementary pathways demonstrate that ASC recycling is supported by an integrated network rather than by MDHAR alone. This metabolic redundancy provides a plausible explanation for the frequently modest phenotypic consequences of MDHAR disruption and helps reconcile the inconsistent effects of MDHAR manipulation on cellular ASC accumulation observed across different species, tissues, and environmental conditions. More broadly, these findings indicate that the physiological functions of MDHAR should be considered within the context of the entire cellular redox network rather than as an isolated component of the ascorbate-glutathione cycle.

## 4 Mechanistic basis of variable ascorbate accumulation following MDHAR perturbation

Extensive biotechnological efforts have focused on manipulating genes of ASC biosynthesis and recycling pathways to elevate ASC concentrations in edible plant organs and improve stress resilience [13,[62], [63], [64]]. Whereas overexpression of enzymes in the l-galactose biosynthetic pathway or DHAR generally leads to predictable increases in ASC content and the ASC/DHA ratio, manipulation of MDHAR produces remarkably inconsistent outcomes across species, tissues, developmental stages, and environmental conditions (Table 2). These contrasting phenotypes challenge the traditional view of MDHAR as a rate-limiting determinant of ASC accumulation and instead suggest that its physiological function is embedded within a highly interconnected and buffered redox network.

**Table 2:** Summary physiological effects of altered MDHAR activity in plants.

Species engineered	Gene source	Enzyme location	Approach	Tissue	Max fold change			Other effects of MDHAR modification/comments
					MDHAR activity	ASC	ASC/DHA	
Arabidopsis	*BrMDHAR* (*Brassica pekinensis*)	n.a.	OE (CMV*35S*)	Leaf	n.a.	n.a.	n.a.	∙ Enhanced low-temperature tolerance [45].
	*BvMDHAR* (*Beta vulgaris*)	n.a.	OE (CMV*35S*)	Leaf	1.1	n.a.	1.4	∙ Increased salt stress tolerance [65].
	*EcMDHAR* (*Eleusine coracana*)	Per	OE (CMV*35S*)	Leaf	2.0	n.a.	n.a.	∙ Increased salt stress tolerance [66].
	*MsMDHAR* (*Medicago sativa*)	Cyt/Per	OE (CMV*35S*)	Leaf	n.a.	1.2	3.5	∙ Higher biomass, more seeds, and larger roots under water stress [67].
Tobacco	*AmMDHAR* (*Avicennia marina*)	Chl	OE (CMV*35S*)	Leaf	2.3	1.7	4.3	∙ Enhanced tolerance to salt stress [68].
	*AtMDHAR1* (*Arabidopsis thaliana*)	Cyt/Per	OE (CMV*35S*)	Leaf	2.1	2.2	3.0	∙ Enhanced tolerance to ozone, salt and polyethylene glycol (PEG) [69].
	*BcMDHAR* (*Brassica campestris*)	Cyt/Chl/Per	OE (CMV*35S*)	Leaf	2.0	0.5	NS	∙ Reduced plant growth [70].
	*MgMDHAR* (*Malpighia glabra*)	Cyt	OE (CMV*35S*)	Leaf	2.1	2.0	3.1	∙ Enhanced tolerance to salt stress [71].
	*SlMDHAR3* (*Solanum lycopersicum*)	Cyt/Per	OE (CMV*35S*)	Leaf	1.3	n.a.	1.2	∙ Enhanced tolerance to salt stress [44].
Tomato ‘Micro-Tom’	*AeMDHAR3* (*Actinidia eriantha*)	Chl	OE (CMV*35S*)	Red fruit	2.0	0.7	NS	∙ 1781 differentially expressed genes identified [72].
	*SlMDHAR3* (*S. lycopersicum*)	Cyt	OE (FMV*34S*)	Leaf	8.0	NS	NS	∙ No ASC elevation in leaf/ripe fruit; reduced ASC in mature green fruit [73].
				Green fruit	n.a.	0.5	n.a.	
				Red fruit	n.a.	NS	n.a.	
Tomato ‘WVa106’	*SlMDHAR3* (*S. lycopersicum*)	Cyt/Per	OE (CMV*35S*)	Leaf	4.3	0.7	0.6	∙ Reduced leaf ASC; no fruit ASC elevation [74].
				Fruit	n.a.	NS	NS	
Tomato ‘Zhongshu4’	*SlMDHAR2* (*S. lycopersicum*)	Chl	OE (CMV*35S*)	Leaf	1.9	1.2	2.2	∙ Improved stress tolerance against temperature, methyl viologen, salt and PEG [75,76].
Rice	*AeMDHAR* (*Acanthus ebracteatus*)	n.a.	OE (CMV*35S*)	Leaf	2.8	4.7	NS	∙ Increased salt tolerance [77].
	*OsMDHAR4* (*Oryza sativa*)	Chl	OE (CMV*35S*)	Leaf	n.a.	1.2	1.7	∙ Enhanced heat sensitivity; reduced stomatal closure [78].
Wheat	*AtMDHAR1* (*A*. *thaliana*)	Cyt/Per	OE (CMV*35S*)	Leaf	n.a.	8.0	n.a.	∙ Increased salt stress [79].
Arabidopsis	*AtMDAHR1*	Per	T-DNA insertion	Leaf	0.4_NADH_	NS	NS	∙ No detectable ASC changes under standard conditions [30]. ∙ Sugar is necessary for germination and early seedling growth of *AtMDAHR4* mutants [47].
					0.5_NADPH_			
	*AtMDAHR2*	Cyt			NS_NADH_	NS	NS	
					0.5_NADPH_			
	*AtMDAHR3*	Cyt			NS _NAD(P)H_	NS	NS	
	*AtMDAHR4*	Per			NS _NAD(P)H_	NS	NS	
Lettuce	*LsMDHAR1*	Chl/Mit	CRISPR/Cas9	Leaf	n.a.	NS	NS	∙ No ASC/phenotypic changes [80].
	*LsMDHAR2/3*	Per						
	*LsMDHAR4*	Cyt/Per						
Mini Chinese cabbage	*BrMDHAR*	n.a.	VIGS	Leaf	0.8	0.8	n.a.	∙ Decreased low-temperature tolerance [45,74].
Rice	*OsMDHAR3*	Cyt	RNAi (Uqi)	Leaf	n.a.	n.a.	0.8	∙ Increased salt sensitivity; reduced biomass/yield [81].
Tomato ‘WV106’	*SlMDHAR3*	Cyt/Per	RNAi (CMV*35S*)	Leaf	0.4	1.2	1.3	∙ Increased ASC; slower growth; reduced yield [74,82].
				Fruit	n.a.	1.2	1.8	
			RNAi (*PPC2*)	Fruit	0.1	n.a.	n.a.	∙ No yield effect [83].
Tomato ‘Moneymaker’			RNAi (CMV*35S*)	Leaf	0.4	NS	NS	∙ No yield effect [83].
Tomato ‘M82’			RNAi (CMV*35S*)	Fruit	0.1	0.8	0.1	∙ Reduced cold storage tolerance [84].
Tomato ‘IL925’				Fruit	0.1	0.7	0.2	
Wheat	*TaMDHAR4*	Per	VIGS	Leaf	n.a.	n.a.	n.a.	∙ Enhanced stripe rust resistance [85].
	*TaMDHAR6*	Chl	VIGS	Leaf	n.a.	n.a.	n.a.	∙ Enhanced stripe rust resistance [86].

Chl, chloroplast; Cyt, cytosol; Per, peroxisome.

OE, overexpression; RNAi, RNA interference; VIGS, Virus-induced gene silencing; CMV*35S*, cauliflower mosaic virus *35S* promoter; F*34S*, figwort mosaic virus constitutive *34S* promoter; PPC2, phosphoenolpyruvate carboxylase 2 promoter; Uqi, ubiquitin promoter.

n.a., not available; NS, no significant changes.

Indeed, increased MDHAR activity does not necessarily enhance ASC accumulation. In several transgenic systems, heterologous expression of MDHAR genes from acerola, Arabidopsis, or tomato elevates total ASC content or maintains a more reduced ASC pool, supporting the canonical role of MDHAR in ASC recycling (Table 2). However, an increasing number of studies report the opposite effect. Overexpression of *SlMDHAR3* reduces ASC accumulation in mature green fruits of tomato cultivar Micro-Tom and in leaves of cherry tomato cultivar WVa106 [73,74]. Likewise, expression of kiwifruit (*Actinidia eriantha*) *AeMDHAR3* in Micro-Tom tomato or *BcMDHAR* from non-heading Chinese cabbage in tobacco decreases endogenous ASC levels rather than increasing them [70,72]. These observations clearly demonstrate that enhancing MDHAR activity alone is insufficient to increase the cellular ASC pool.

One explanation for these contrasting responses is that ASC concentration is determined by the dynamic balance between biosynthesis, oxidation, recycling, degradation, and transport rather than by recycling capacity alone [87]. Increasing MDHAR activity may accelerate ASC regeneration, but this effect can be offset if ASC consumption or turnover increases simultaneously. Consistent with this model, reduced ASC accumulation in *BcMDHAR*-overexpressing tobacco and *AeMDHAR3*-expressing Micro-Tom tomato is accompanied by elevated expression of APX, suggesting that enhanced ASC oxidation counteracts increased recycling [70,72]. However, *APX* expression remains unchanged in *SlMDHAR3*-overexpressing WVa106 plants [74], indicating that altered antioxidant consumption alone cannot fully explain the observed phenotypes. Additional regulatory processes must therefore contribute to the context-dependent relationship between MDHAR activity and ASC homeostasis.

Genetic background appears to be one such determinant. Tomato provides a particularly illustrative example. Overexpression of chloroplast-localized *SlMDHAR2* in the large-fruited cultivar Zhongshu 4 significantly increases leaf ASC content and nearly doubles the ASC/DHA ratio [75]. In contrast, increased MDHAR activity consistently suppresses ASC accumulation in the small-fruited cultivars Micro-Tom and WVa106 [[72], [73], [74]] (Table 2). These contrasting responses indicate that the metabolic consequences of MDHAR manipulation are strongly influenced by genotype rather than by enzyme activity alone.

Feedback regulation of ASC biosynthesis probably contributes to this variation. Translation of GDP-l-galactose phosphorylase (GGP), the principal rate-limiting enzyme in ASC biosynthesis, is negatively regulated by ASC through an upstream open reading frame, thereby providing a feedback mechanism that stabilizes the ASC pool [88]. Consistent with this model, *GGP* expression decreases in *BcMDHAR*-overexpressing tobacco [70]. Nevertheless, biosynthetic feedback alone cannot account for all observed phenotypes. For example, in Micro-Tom tomato, overexpression of *SlMDHAR3* reduces ASC accumulation only during the mature green stage, whereas ASC levels remain unchanged in red-ripe fruit [73]. These developmental differences suggest that the impact of MDHAR is further shaped by tissue-specific metabolic programs and changing physiological demands during fruit development.

Reverse genetic studies reinforce the conclusion that MDHAR functions in a highly context-dependent manner. Under standard growth conditions, simultaneous disruption of all five *AtMDHAR* genes in Arabidopsis or all four *LsMDHAR* genes in lettuce (*Lactuca sativa*) produces little or no change in total ASC content or cellular redox status, demonstrating substantial functional redundancy within the MDHAR family [30,80]. Similarly, constitutive silencing of *SlMDHAR3* in tomato cultivar Moneymaker has no detectable effect on ASC levels in either leaves or fruits [83]. By contrast, *Osmdhar3* mutants in rice, virus-induced silencing of *BrMDHAR* in mini Chinese cabbage, and *SlMDHAR3* RNA interference lines in tomato cultivars M82 and IL925 all exhibit reduced ASC content and a more oxidized ASC pool [45,81,84]. Even within tomato, the consequences of suppressing *SlMDHAR3* differ depending on the spatial pattern of gene silencing. Whole-plant RNA interference in WVa106 increases ASC accumulation in both leaves and fruits but markedly reduces fruit yield, whereas fruit-specific suppression has little effect on productivity [83]. These findings indicate that leaf MDHAR activity exerts a stronger influence on source strength and reproductive performance than fruit-localized MDHAR in this genetic background.

Collectively, current evidence argues against a simple linear relationship between MDHAR activity and cellular ASC accumulation. Instead, MDHAR should be viewed as a component of an integrated redox regulatory network in which ASC recycling is coordinated with biosynthetic feedback, antioxidant consumption, alternative MDHA-reducing pathways, developmental regulation, and source-sink interactions. Therefore, the magnitude and direction of ASC responses following MDHAR perturbation are determined by a combination of genetic background, isoform-specific properties, subcellular localization, developmental stage, and tissue-specific metabolic demands, rather than by MDHAR activity alone. This network-based perspective provides a mechanistic framework for reconciling apparently contradictory phenotypes across species and highlights the importance of considering system-level regulation when exploiting MDHAR for crop biofortification and stress-resilience engineering.

## 5 MDHAR in stress tolerance, plant growth and developmental regulation

### 5.1 Dual roles in abiotic and biotic stress tolerance

As a central component of the ascorbate-glutathione cycle, MDHAR plays a pivotal role in plant acclimation to environmental challenges by sustaining ASC regeneration and maintaining cellular redox balance. In many cases, enhanced MDHAR activity promotes ASC recycling, restricts excessive ROS accumulation, and consequently improves tolerance to diverse abiotic and biotic stresses (Table 2). Numerous transgenic studies support this protective function. For example, heterologous expression of MDHAR genes from sugar beet (*Beta vulgaris*) and finger millet (*Eleusine coracana*) enhances salt tolerance in Arabidopsis [65,66]. Similarly, overexpression of *AtMDHAR1* in tobacco maintains higher net photosynthetic rates under ozone, salinity, and polyethylene glycol (PEG)-induced osmotic stress, while preserving the effective quantum yield of photosystem II (PSII) under ozone and salt exposure [69]. Chloroplast-targeted overexpression of *SlMDHAR2* in tomato cultivar Zhongshu 4 also enhances tolerance to multiple oxidative challenges, including heat, chilling, methyl viologen-induced oxidative stress, salinity, and PEG treatment [75,76]. Beyond abiotic stress adaptation, MDHAR contributes to plant immunity. In Chinese cabbage (*B*. *rapa*), the receptor-like protein BrRLP1 physically interacts with BrMDHAR1 to enhance resistance against downy mildew, providing direct evidence that ASC recycling is functionally connected with immune signaling pathways [89].

However, accumulating evidence indicates that the contribution of MDHAR to stress tolerance is not universally positive. In some contexts, MDHAR functions as a negative regulator of stress responses, revealing a more complex physiological role than previously appreciated. Rice plants overexpressing *OsMDHAR4* maintain elevated ASC levels and a higher ASC/DHA ratio before and after heat stress, yet display increased heat sensitivity [78]. The failure of enhanced antioxidant capacity to alleviate heat susceptibility suggests that the function of *OsMDHAR4* extends beyond regulation of ASC metabolism and may involve additional redox-dependent or redox-independent signaling mechanisms.

Recent genetic studies further support the emerging view that MDHAR acts as a regulator of redox signaling rather than solely as an ROS-scavenging component. Although none of the six Arabidopsis MDHAR isoforms is individually required for vegetative growth under non-stress conditions [19,30], loss of *MDHAR2* suppresses salicylic acid (SA) accumulation and lesion formation in the oxidative stress-sensitive *cat2* background [30]. In *cat2* plants, peroxisome-derived H_2_O_2_ triggers glutathione accumulation and activates SA-dependent defense signaling. The attenuation of these responses in *cat2 mdhar2* mutants indicates that the cytosolic ascorbate-glutathione cycle serves as an important signaling relay that integrates oxidative information originating from other cellular compartments. Consistently, simultaneous disruption of *DHAR1*, *DHAR2*, and *DHAR3* also suppresses SA accumulation and lesion development in *cat2*, accompanied by reduced glutathione oxidation (lower GSSG accumulation) [90]. By contrast, individual disruption of the peroxisomal isoforms *AtMDHAR1* or *AtMDHAR4* has little effect on the ascorbate pool but enhances glutathione oxidation under *cat2*-induced oxidative stress [30], suggesting functional redundancy between these two isoforms. Generation and characterization of a *cat2 mdhar1 mdhar4* triple mutant will therefore be necessary to determine whether the peroxisomal MDHAR isoforms collectively contribute to oxidative signal transduction.

Consistent with the context-dependent nature of MDHAR function, reducing *MDHAR* expression can also enhance stress tolerance in certain genetic backgrounds. Silencing *TaMDHAR4* or *TaMDHAR6* significantly improves wheat resistance to stripe rust (*Puccinia striiformis* f. sp. *tritici*), indicating that these isoforms negatively regulate pathogen defense [39,86]. Likewise, loss of *OsMDHAR4* enhances rice thermotolerance by promoting H_2_O_2_-dependent stomatal closure [78], whereas the *Atmdhar6* mutant exhibits increased tolerance to the environmental pollutant TNT [52]. Together, these findings demonstrate that MDHAR is not intrinsically beneficial or detrimental for stress resistance. Rather, its physiological consequences depend on cellular context, isoform-specific functions, subcellular localization, and interactions with redox signaling, hormone pathways, and stress-specific regulatory networks.

### 5.2 Roles in plant growth and developmental programming

Beyond stress adaptation, MDHAR contributes to diverse aspects of plant growth and development (Table 2). One of the earliest indications of its developmental importance came from characterization of the Arabidopsis *sdp2* mutant, in which impaired endogenous MDHAR activity causes seedling lethality under photoautotrophic conditions. This phenotype is fully rescued by exogenous sugar supplementation, demonstrating that MDHAR-mediated redox regulation is required for the transition from heterotrophic to autotrophic growth [47]. This finding highlights the close relationship between ASC metabolism, photosynthetic establishment, and developmental progression.

Increasing evidence further links MDHAR-dependent redox homeostasis with organ development and crop productivity. In cotton, long- and medium-fiber cultivars exhibit higher MDHAR activity, increased ASC content, and a more reduced ASC/DHA ratio than short-fiber cultivars, suggesting that efficient ASC recycling supports fiber cell elongation by maintaining an appropriate redox environment [20]. During pepper fruit ripening, plastid differentiation from chloroplasts to chromoplasts is accompanied by a three-to six-fold increase in plastidial MDHAR activity, indicating a potential role for ASC recycling during plastid remodeling and fruit maturation [91]. Similarly, whole-plant silencing of *SlMDHAR3* in cherry tomato cultivar WVa106 markedly reduces fruit yield under both optimal and carbon-limited conditions, demonstrating that MDHAR contributes to source strength, carbon allocation, and reproductive productivity [82]. Conversely, heterologous expression of *BcMDHAR* from non-heading Chinese cabbage in tobacco results in severe growth retardation [70], further emphasizing that the developmental consequences of MDHAR manipulation depend strongly on species background, isoform identity, and metabolic context.

Taken together, these studies demonstrate that MDHAR functions beyond its classical role in antioxidant protection. By integrating ASC metabolism with photosynthetic performance, carbon allocation, developmental transitions, and reproductive growth, MDHAR represents a critical interface between cellular redox status and plant developmental programs. Future research should focus on identifying the molecular mechanisms through which specific MDHAR isoforms connect redox metabolism with developmental signaling pathways, thereby enabling more precise manipulation of MDHAR activity for crop improvement.

## 6 Conclusions and perspectives

MDHAR occupies a central position in the plant redox network by connecting ROS detoxification, ASC recycling, and redox signaling. Rather than functioning merely as an antioxidant enzyme, accumulating evidence indicates that MDHAR is a multifunctional regulator that coordinates cellular redox homeostasis with stress adaptation, metabolic reprogramming, and plant development. Depending on physiological and environmental conditions, MDHAR either scavenges excessive ROS to protect cellular integrity or fine-tunes ROS levels to preserve signaling competence during growth, development, and stress responses. Moreover, emerging evidence that MDHAR activity is modulated by reactive nitrogen species (RNS) and potentially reactive sulfur species (RSS; Fig. 2) underscores extensive crosstalk among ROS-, RNS-, and RSS-dependent signaling pathways [92], positioning MDHAR as a key node within an integrated cellular redox regulatory network.

Despite substantial progress, several fundamental questions remain unresolved. First, the molecular mechanisms through which MDHAR influences ASC homeostasis are still incompletely understood. The highly variable effects of MDHAR overexpression or loss of function on ASC accumulation across species, tissues, developmental stages, and environmental conditions indicate that MDHAR functions within a dynamic network integrating ASC biosynthesis, recycling, degradation, transport, and redox signaling, rather than acting as an independent rate-limiting determinant of ASC content. Identifying the regulatory components and metabolic interactions that govern these divergent outcomes should therefore be a major priority for future research.

Second, the physiological specialization of individual MDHAR isoforms remains poorly defined. Their distinct subcellular localizations strongly suggest compartment-specific functions in maintaining organellar redox homeostasis, yet how chloroplastic, mitochondrial, cytosolic, and peroxisomal isoforms cooperate to coordinate whole-cell redox balance remains largely unknown. Resolving the functional interplay among these compartmentalized isoforms will be essential for understanding how redox information is integrated across cellular compartments.

Third, although multiple PTMs of MDHAR have been identified, their mechanistic significance remains largely unexplored. For example, *S*-nitrosylation activates MDHAR in mini Chinese cabbage but inhibits the peroxisomal enzyme in pea, demonstrating that the functional consequences of PTMs are highly dependent on species, isoform, and cellular context. Defining the structural determinants and signaling mechanisms underlying these contrasting responses will be critical for understanding the dynamic regulation of MDHAR activity under fluctuating environmental conditions.

Finally, the physiological functions of MDHAR almost certainly extend beyond canonical ASC recycling. Its ability to reduce non-canonical substrates, including phenoxyl radicals and TNT (Fig. 3A), points to broader roles in secondary metabolism, xenobiotic detoxification, and environmental adaptation. Whether additional endogenous substrates, interacting proteins, or protein complexes contribute to these non-canonical functions remains an important question for future investigation.

Addressing these challenges will require multidisciplinary approaches integrating genetics, structural biology, systems biology, and crop biotechnology. Higher-order genome editing combined with transcriptomics, proteomics, metabolomics, and spatially resolved redox profiling will facilitate dissection of isoform-specific functions and regulatory networks. Complementary structural approaches, including cryo-electron microscopy, artificial intelligence-assisted protein structure prediction, and quantitative biochemical analyses, should further elucidate the molecular basis of substrate recognition, cofactor specificity, and PTM-mediated regulation. Equally important will be defining how MDHAR interacts with complementary redox systems, including CYB561 proteins, DOMON-domain proteins, TRXs, glutaredoxins, and other antioxidant pathways, to establish a systems-level framework for cellular redox regulation.

From a translational perspective, MDHAR represents a promising yet challenging target for crop improvement. The highly variable outcomes of manipulating individual *MDHAR* genes suggest that future engineering strategies should move beyond single-gene approaches. Instead, coordinated engineering of MDHAR together with key ASC biosynthetic genes, such as *GGP* and other enzymes of the l-galactose pathway [87,93], or with complementary antioxidant pathways [94], is likely to provide a more effective strategy for simultaneously enhancing ASC accumulation, stress resilience, and crop productivity. In addition, plastid genome engineering offers an attractive platform for coordinated expression of multiple antioxidant genes because of its high transgene expression, stable inheritance, and exceptional capacity for multigene stacking [95,96]. Systematic evaluation of these combinatorial engineering strategies under agronomically relevant field conditions will be essential for translating mechanistic insights into practical applications. Notably, tomato fruit with exceptionally high ASC accumulation exhibited impaired flower development and produced seedless fruit [97], suggesting that fine-tuning ASC abundance, rather than maximizing its accumulation, is critical for balancing nutritional quality, stress tolerance, and reproductive yield in crop breeding.

In conclusion, MDHAR is emerging not simply as an enzyme of the ascorbate–glutathione cycle, but as a multifunctional hub that integrates antioxidant metabolism with redox signaling, stress adaptation, and developmental regulation. Future studies that combine mechanistic, structural, and systems-level analyses with translational crop engineering will not only advance our understanding of plant redox biology but also establish a foundation for designing crops with enhanced nutritional value, improved environmental resilience, and greater agricultural productivity under a changing climate.

## CRediT authorship contribution statement

**Yuxin Xiao:** Writing – original draft. **Jiatong Zhou:** Writing – original draft. **Shengchun Li:** Writing – review & editing, Supervision, Conceptualization.

## Declaration of competing interest

The authors declare no competing interests.

## Data Availability

No data was used for the research described in the article.
